# Supplementary material for: Predicting locations of cryptic pockets from single protein structures using the PocketMiner graph neural network
Source: Nat Commun. 2023 Mar 1;14:1177. doi: 10.1038/s41467-023-36699-3 (PMC9977097; doi:10.1038/s41467-023-36699-3)
Supplement: Supplementary file 1 — Supplementary Information [file 41467_2023_36699_MOESM1_ESM.pdf]

Supplementary Information

Predicting locations of cryptic pockets from single protein structures using the PocketMiner graph neural network

Artur Meller[1,2*], Michael Ward[1*], Jonathan Borowsky[1], Meghana Kshirsagar[3], Jeffrey M. Lotthammer[1], Felipe Oviedo[3], Juan Lavista Ferres[3], Gregory R. Bowman[1,4,‡]

1 Department of Biochemistry and Molecular Biophysics, Washington University in St. Louis, 660 S. Euclid Ave., Box 8231, St. Louis, MO 63110 2 Medical Scientist Training Program, Washington University in St. Louis, 660 S. Euclid Ave., St. Louis, MO 3 AI for Good Research Lab, Microsoft, Redmond, WA 4 Department of Biochemistry and Molecular Biophysics, University of Pennsylvania, 3620 Hamilton Walk, Philadelphia, PA, 19104

*Authors contributed equally

ǂ Corresponding author (grbowman@seas.upenn.edu)

Supplementary Figures

Contents

1. Project outline with comparison of different prediction tasks
2. Featurization of TEM-1 β-lactamase
3. Label consistency in independent simulations
4. RMSD distributions
5. Ligands of carbonic anhydrase
6. Curation of negative examples
7. Structural alignments of high sequence identity proteins
8. PocketMiner predictions on the test set
9. PocketMiner predictions for Wnt2 and simulations of Wnt2
10. 3D CNN grid featurization
11. 3D CNN training and validation PR-AUC

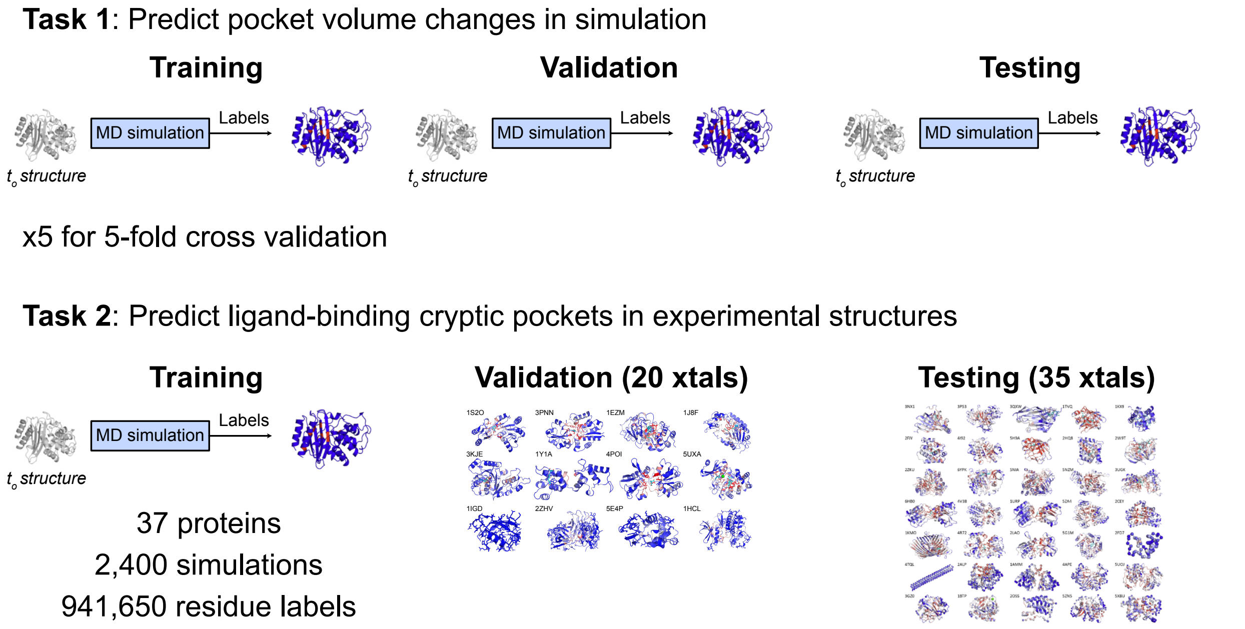

Supplementary Figure 1. **Outline of project with comparison of different prediction tasks.** Initially, we assessed if deep learning models could make accurate inferences about simulations based on starting structures in task 1. We also compared different deep learning architectures (i.e., GVP-GNN vs. 3D-CNN). For task 1, validation and testing were performed with molecular dynamics-derived labels (see Methods). Next, we assessed if models trained with simulation labels could accurately predict where ligand-binding cryptic pockets are found in experimental structures. We used a validation set to evaluate different labeling schemes. Finally, we tested on a collection of experimental structures that included ligand-free experimental structures known to rearrange into cryptic pockets upon ligand binding as well as negative examples (see Methods).

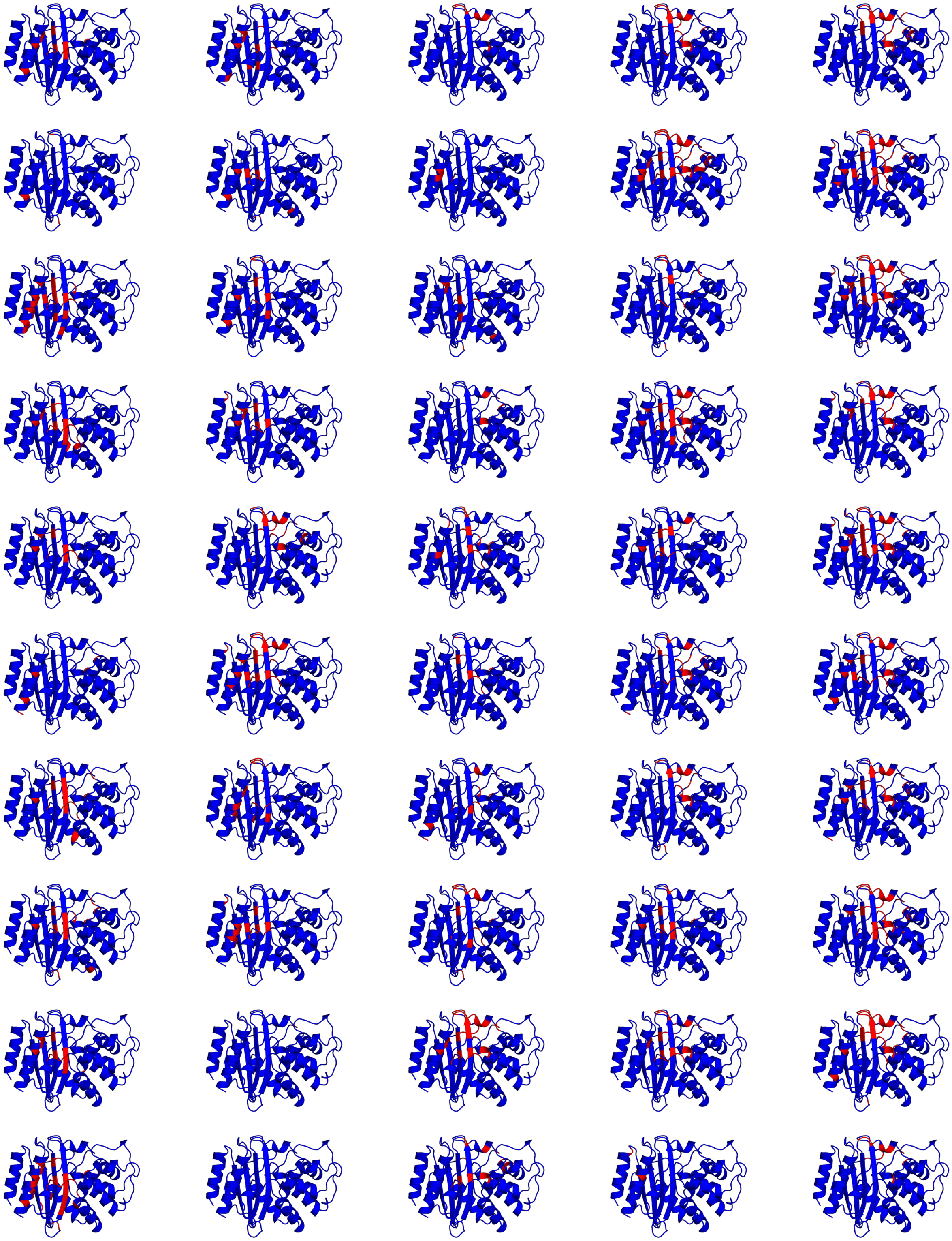

Supplementary Figure 2. **Featurization of TEM-1 β-lactamase.** Featurization repeatedly identifies residues of both the horn site and omega loop pockets as cryptic sites without identifying residues in most other areas of the protein. Residues with negative featurization

labels are colored blue while those with positive labels are colored red. Labels from 5 rounds (columns) of FAST with 10 parallel simulations each (rows) are shown projected onto the *apo* crystal structure used as a starting structure, with subsequent rounds running from left to right.

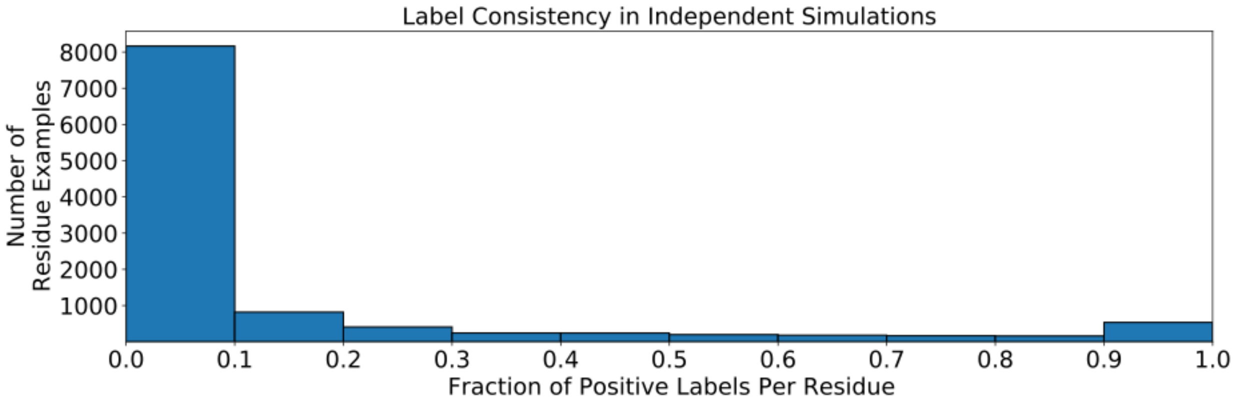

Supplementary Figure 3. **Label consistency in independent simulations.** We determined the fraction of times that each residue participates in a cryptic pocket opening across independent 40 ns-long simulations launched from the same starting structure. Values of 0 and 1 indicate perfect consistency (either opening in no simulations or opening in all simulations). The two peaks at 0 and 1 indicate that our labeling scheme produces consistent labels across independent simulations launched from the same structure. However, among positive labels, a positive example was more likely to only open in a minority of independent simulations, indicating that many pocket opening events are rare on the timescales analyzed (40 ns).

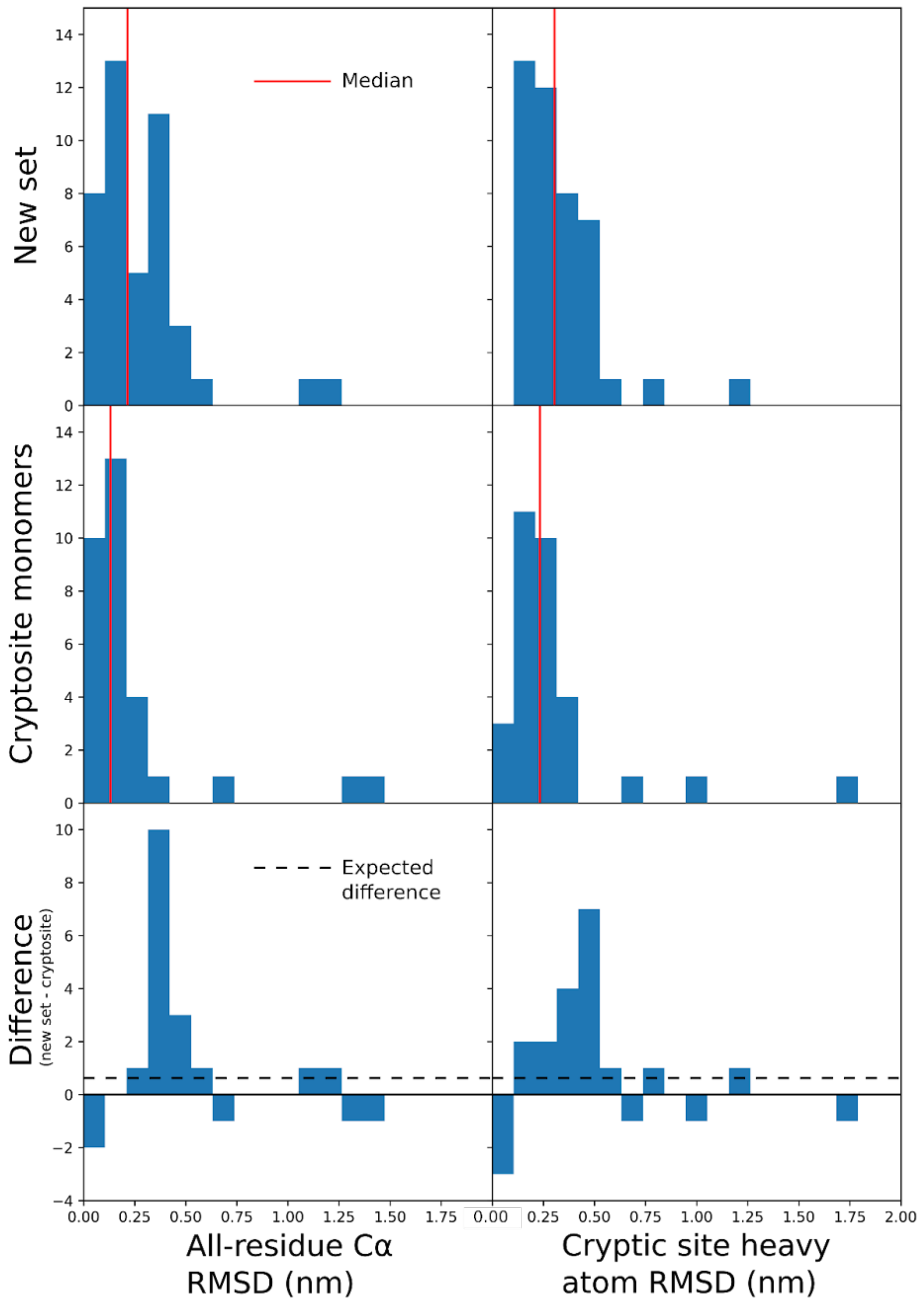

Supplementary Figure 4. **RMSD distributions for the new protein set compiled in this work and for physiological monomers in the CryptoSite set without large gaps.** Medians are shown in red.

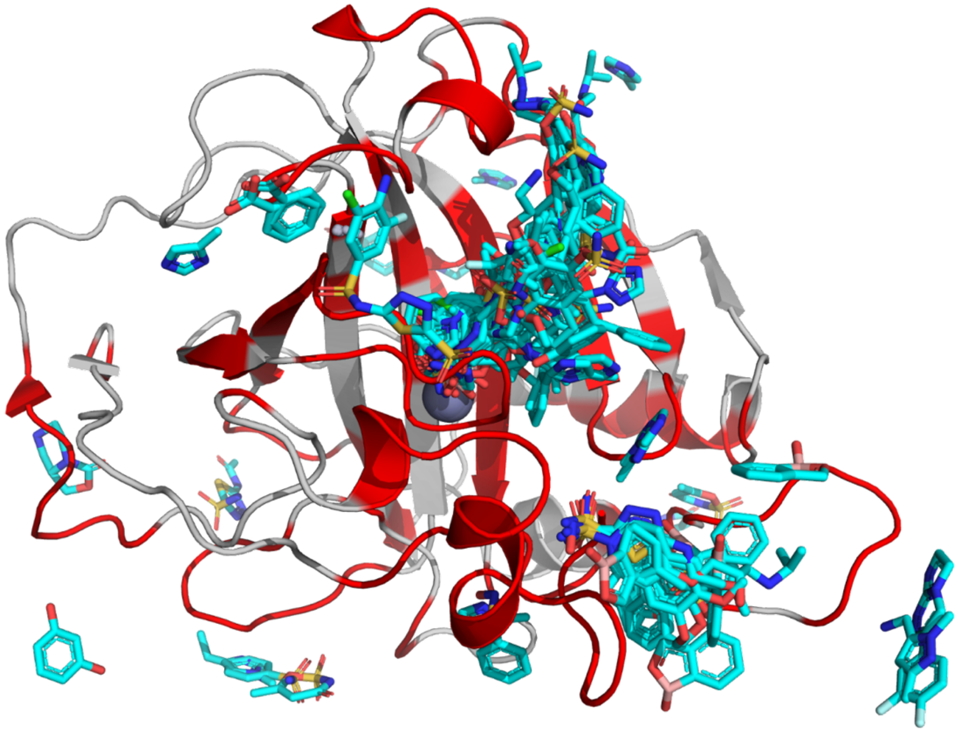

Supplementary Figure 5. **Ligands binding carbonic anhydrase.** The carbonic anhydrase cluster centroid (PDB ID 1YDA) is shown as a ribbon. Residues which are within 5Å of a valid MOAD ligand in any centroid are shown in red, while the remainder, which were used as negative true labels for testing and validation, are shown in white. A subset of ligands which are collectively within 5Å of every nonnegative residue are shown as cyan sticks.

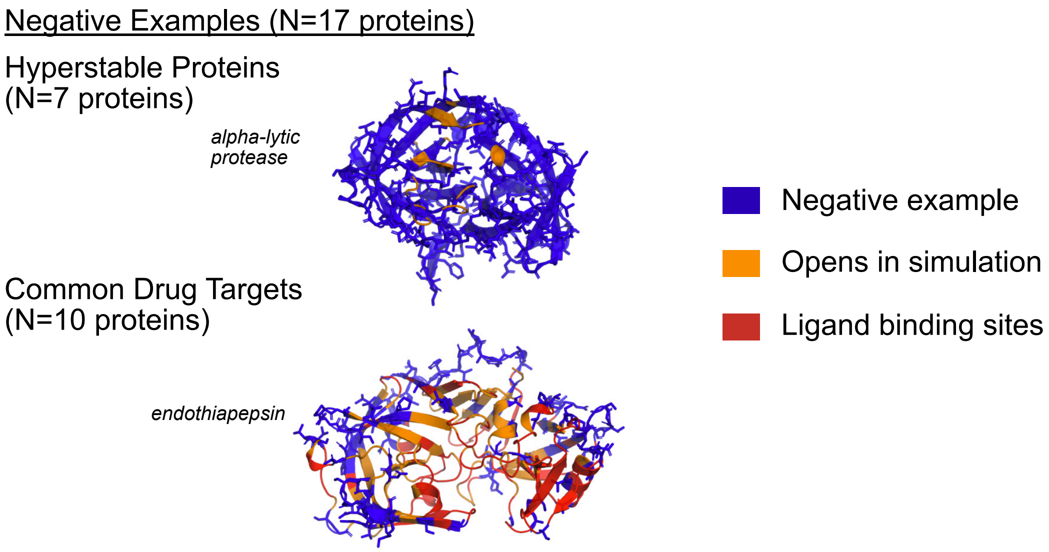

Supplementary Figure 6. **Negative examples were curated from hyper-rigid proteins and common drug targets.** We ran simulations of both kinds of proteins to determine if any pockets formed during these simulations. Any residues that were adjacent to a pocket in simulation were excluded from the test set. For common drug targets, any residues that bind a ligand were also excluded from the test set.

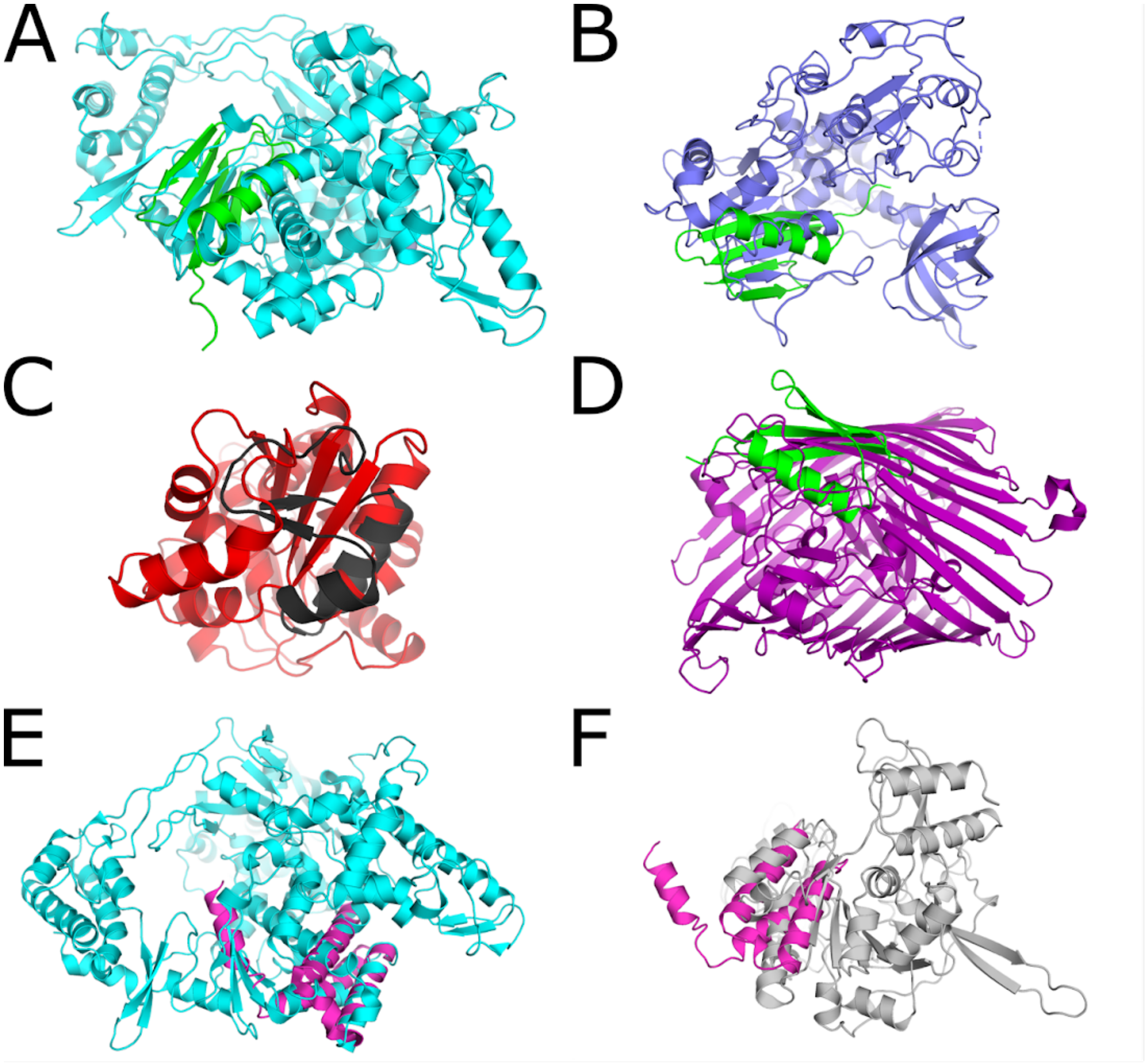

Supplementary Figure 7. **Structural alignments of high-sequence-identity pairs of proteins used in this paper**, using PyMol's *cealign* function (see Supplementary Table 2).
A. SARS-CoV-2 nsp12 (cyan) and PDB ID 1IGD (green). 1IGD is ~1/10th the length of nsp12. It aligns reasonably well to one area of nsp12, but the beta strand polarity and topology of the loops connecting the beta strands does not match.
B. PDB ID 1IGD and SARS-CoV-2 nsp13 (blue). Once again, 1IGD aligns reasonably well to one area of the much larger nsp13, but the beta strand polarity and topology of the loops connecting the beta strands does not match.
C. PDB ID 2FD7 (black) and PDB ID 2OHG (red), showing no meaningful structural homology.
D. PDB ID 1IGD and PDB ID 1KMO (purple), showing no meaningful structural homology.
E. SARS-CoV-2 nsp12 and SARS-CoV-2 nsp7 (magenta), showing no meaningful structural homology.
F. PDB ID 5NZM (grey) and SARS-CoV-2 nsp7, showing no meaningful structural homology.

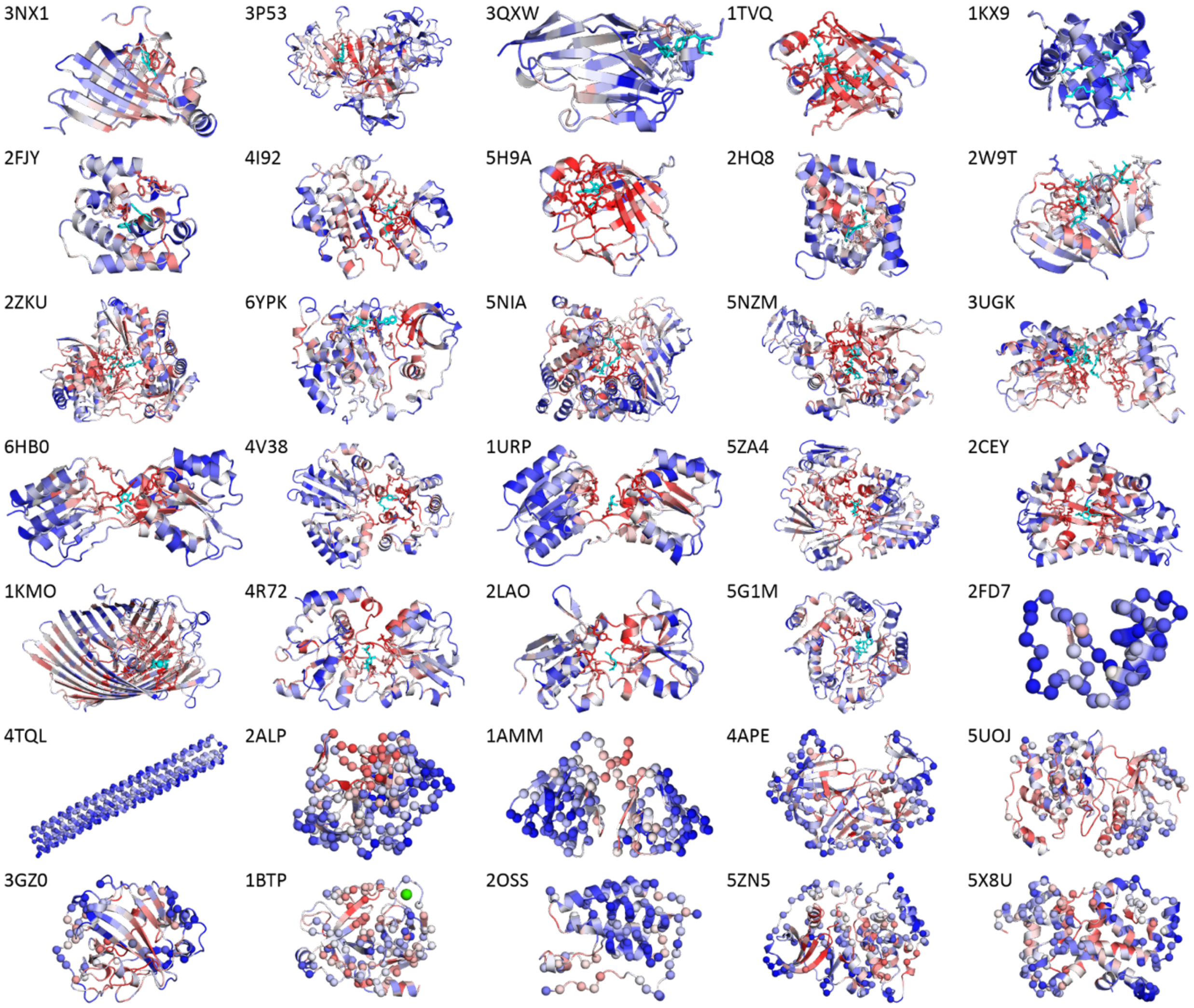

Supplementary Figure 8. **PocketMiner predictions on the test set.** Proteins are shown as ribbons, colored from blue to red as predictions range from negative to positive. Ligand-lining residues (positive true labels) are shown as sticks. Residues in proteins believed to lack cryptic pockets which did not line pockets in simulation and residues in well-studied proteins which were neither adjacent to drug-like ligands nor lined pockets in simulations (negative true labels) are shown in spheres. Protein-bound ions are shown as spheres and colored according to their respective elements. Ligands binding in cryptic pockets are shown in cyan sticks (and cyan spheres for the two iron ions which comprise part of PDB ID 1KMO's cryptic ligand assembly). Proteins are ordered left to right and then top to bottom, with cryptic pocket examples first in order of decreasing difference between the mean distance between ligand-lining residues in *holo* and *apo* (see attached SI spreadsheet tab validation_and_test_sets, columns O-R). Forward pockets are listed first, followed by ones involving a mixture of forward and reverse motions, followed by reverse pockets. 2FJY is listed out of order and placed next to the functionally related protein 1KX9 because the large number of *apo* residues which become unresolved in *holo* render the assignment of pocket direction unreliable. After the cryptic pocket

examples are three proteins believed to be highly rigid, followed by proteins with many *holo* crystal structures.

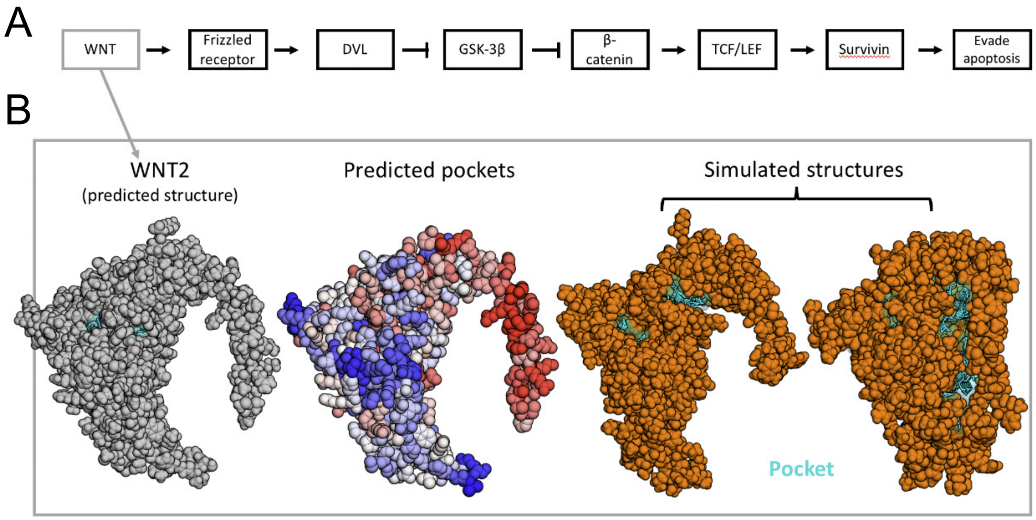

Supplementary Figure 9. **PocketMiner predicts a cryptic pocket in Wnt2 that opens in simulation.** A) Wnt2 is part of the Wnt2 signal transduction pathway that regulates apoptosis and has been identified as a cancer target. B) PocketMiner predicts a cryptic pocket will form based on the AlphaFold-predicted structure of Wnt2. In simulation, a cryptic pocket forms as a result of an interdomain closure.

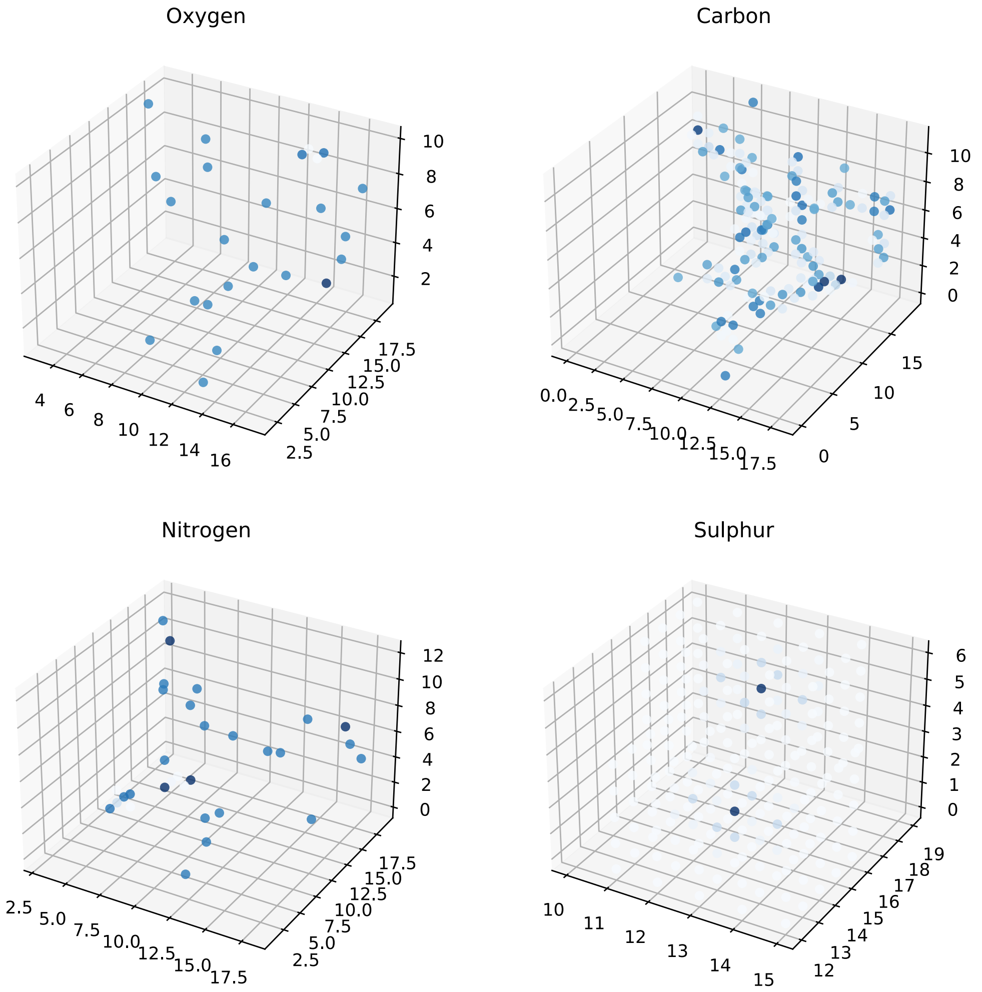

Supplementary Figure 10. **Schematic depicting 3D grid featurization for 3D-convolutional neural networks.** There are four separate channels, one for each of the elements shown above.

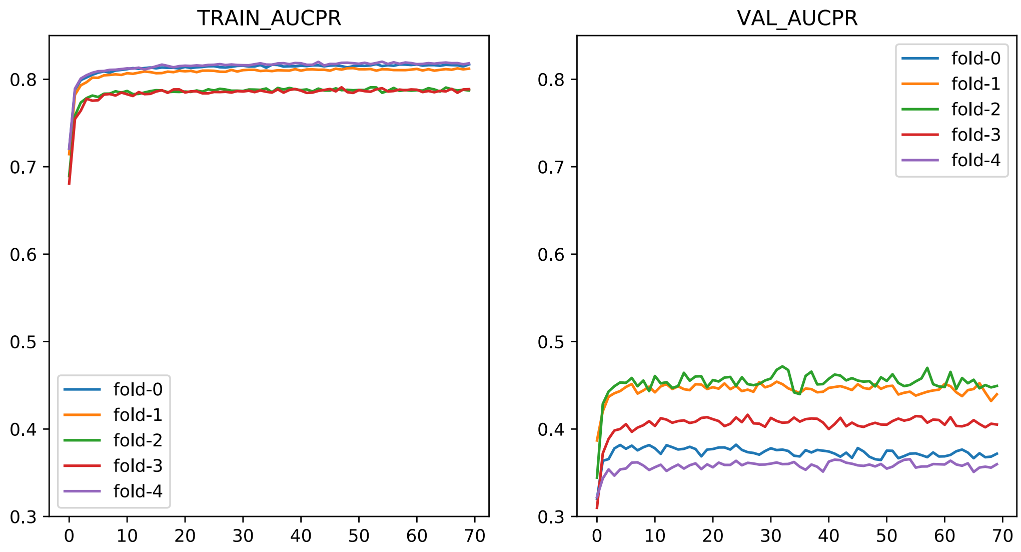

Supplementary Figure 11. **3D convolutional neural network training and validation PR-AUC as a function of epoch** (x-axis) demonstrate convergence around 10 epochs.

Supplementary Tables

Contents

1. Performance comparison between GVP-GNN and 3D-CNN on task 1 test sets
2. GVP-GNN model parameter scan results for a task 1 validation set
3. Comparison of GVP-GNN performance across different class balancing schemes and batch sizes on task 1 validation sets
4. Comparison of 3D-CNN performance across different hyperparameters on task 1 validation sets
5. Highest sequence identities among proteins in this work
6. Performance of LIGSITE volume changes in simulation at ligand-binding cryptic pocket prediction
7. FAST simulations
8. PocketMiner and CryptoSite protein-level performance
9. PocketMiner sensitivity by protein topology and cryptic pocket type

Supplementary Table 1: Performance comparison between GVP-GNN and 3D-CNN on task 1 test sets (predicting pocket volume changes in simulation from starting structure). We used 5-fold cross-validation where the overall dataset was split into 5 groups by protein. 3 folds were used for training; 1 fold was used for validation; and 1 fold was used for testing. We optimized hyperparameters (i.e., batch size, class balancing scheme, network parameters like dropout) separately for each split using the validation set (see Tables S2-5).

Split	GVP-GNN Test PR-AUC	3D-CNN Test PR-AUC
0	0.32	**0.36**
1	**0.40**	0.36
2	**0.48**	0.45
3	**0.64**	0.49
4	0.38	**0.39**

Supplementary Table 2: GVP-GNN model parameter scan results for a task 1 (predicting pocket volume changes in simulations based on starting structures) validation set show sensitivity to the choice of learning rate. This scan was performed using a single validation fold (fold 1), and the optuna library was used to select parameters across a range of options. We set a low learning rate (2e-5) and kept the default GVP-GNN parameters from the original publication for all subsequent experiments.

Learning Rate	Dropout	GVP hidden scalar dimension	Validation PR-AUC
8.00E-05	0.07	50	0.288
6.00E-05	0.06	75	0.245
2.20E-04	0.06	100	0.238
9.00E-05	0.12	50	0.205
8.00E-05	0.27	100	0.204
5.30E-04	0.06	50	0.192
3.80E-04	0.12	75	0.144
8.70E-04	0.20	50	0.067
4.50E-04	0.20	100	0.066
7.80E-04	0.09	50	0.065

Supplementary Table 3: Comparison of GVP-GNN performance across different class balancing schemes and batch sizes on task 1 validation sets. We used 5-fold cross-validation where the overall dataset was split into 5 groups by protein (3 folds for training, 1 for validation, 1 for testing). In the table below, we compare performance for different training parameters (i.e., batch size and class balancing scheme) separately for each split using the validation set. Generally, smaller batch sizes contributed to better performance. Bolding is used to indicate the best training setup for each split.

Because positive label fraction across all labels is 0.1, the following weighting and class balancing schemes were used:

- none
- weighting: loss was weighted by the inverse proportion of negative and positive examples in each batch
- oversampling: minor class (usually positives) was oversampled in each batch
- undersampling: major class (usually negatives) was undersampled in each batch
- constant size balancing: the same number of positive and negative examples was used for each batch in a 1:1 ratio

Intermediate labels were those with volume changes between 116 and 20 LIGSITE grid points.

**Parameters**				**PR-AUC on task 1 validation set**				
**intermediate labels included as negatives**	**training label weighting or sampling scheme**	**number of residues drawn**	**Batch size**	**Split**				
				**0**	**1**	**2**	**3**	**4**
no	none	n/a	1 prot	0.398	0.348	0.285	0.270	0.247

yes	none	n/a	1 prot	0.224	0.457	0.599	0.362	0.252
no	undersampling	n/a	1 prot	0.322	0.368	0.319	0.275	0.325
yes	undersampling	n/a	1 prot	0.352	0.377	0.587	0.357	0.321
no	oversampling	n/a	1 prot	0.428	**0.487**	0.281	0.372	0.344
yes	oversampling	n/a	1 prot	0.316	0.337	0.279	0.307	0.221
no	weighting	n/a	1 prot	0.335	0.240	0.459	0.257	0.271
yes	weighting	n/a	1 prot	0.406	0.318	0.422	0.267	0.215
no	none	n/a	32 resis	0.373	0.378	0.481	0.280	0.333
yes	none	n/a	32 resis	0.308	0.334	0.425	0.274	0.337
no	weighting	n/a	32 resis	0.203	0.458	0.587	0.394	0.279
yes	weighting	n/a	32 resis	0.388	0.385	0.635	0.410	0.263
no	constant size balancing	160	32 resis	0.355	0.386	0.488	0.243	0.312
yes	constant size balancing	n/a	32 resis	0.284	0.378	0.523	0.368	0.246
no	none	n/a	4 resis	**0.480**	0.399	0.555	0.398	0.355
no	constant size balancing	n/a	4 resis	0.464	0.467	0.497	0.381	**0.359**
yes	constant size balancing	n/a	4 resis	0.316	0.473	0.579	0.377	0.338
no	none	n/a	4 resis	0.458	0.361	0.582	0.360	0.322
yes	none	160	4 resis	0.353	0.487	**0.648**	**0.491**	0.315
yes	none	n/a	4 resis*	0.331	0.396	0.621	0.406	0.284
yes	none	160	4 resis	0.394	0.426	0.530	0.358	0.298
yes	constant size balancing	160	4 resis	0.410	0.437	0.496	0.420	0.348

*4 residues were drawn from 4 different proteins randomly in this training setup. Otherwise, 4 residues were drawn from a single protein.

Supplementary Table 4: Comparison of 3D-CNN performance across different hyperparameters on task 1 validation sets.

Network changes (if any)	**Class-balancing**	**Batch-size***	**Learning-rate**	**Drop-out prob.**		**Validation AUC-PR**				
						**Split**				
						**0**	**1**	**2**	**3**	**4**
	1:1	32	0.0001	0.7		0.288	**0.253**	**0.500**	0.472	**0.313**
	1:2	32	0.0001	0.7		**0.297**	0.250	0.500	**0.482**	0.310
	None	32	0.0001	0.7		0.230	0.233	0.424	0.433	0.277
	1:1	64	0.0001	0.7		0.212	0.235	0.472	0.466	0.271
	1:2	64	0.0001	0.7		0.295	0.242	0.417	0.477	0.266
	1:2	32	0.001	0.7		0.172				
	1:2	32	0.01	0.7		0.277				
	1:1	32	0.0001	0.1		0.091	0.104	0.303	0.274	0.101
	1:2	32	0.0001	0.1		0.091	0.100			
	1:2	32	0.0001	0.5		0.225	0.185			
	1:2	32	0.0001	0.3		0.147	0.149			
	1:1	128	0.001	0.7		0.231				
	None	128	1.00E-05	0.7		0.171				
4 layers	1:2	128	1.00E-05	0.7		0.219				
4 layers	1:2	128	0.001	0.7		0.219				
	1:2	128	0.0001	0.1		0.071				
	1:2	128	1.00E-05	0.1		0.090				
	1:2	128	1.00E-05	0.3		0.135				
	None	1 protein	1.00E-05	0.7		0.146				
	1:2	1 protein	1.00E-05	0.7		0.234				
	1:2	1 protein	0.001	0.7		0.208				

*units of residues unless explicitly labeled

Supplementary Table 5: Highest sequence identities among proteins in this work. *Apo* PDB IDs are given where applicable, and the PBD IDs of SARS and MERS protein structures (or those used for homology modeling) can be found in the training_5_fold_cv tab of the attached SI spreadsheet. The complete list of sequence identities between proteins used in this study exceeding 40% can be found in the sequence_identity tab of the same spreadsheet.

**Protein 1**	**Protein 2**	**Percent identity**	**Notes**
SARS-1-nsp16	SARS-2-nsp16	93.493	All in the same 5-fold cross validation fold
MERS-nsp16	SARS-2-nsp16	65.870	
MERS-nsp16	SARS-1-nsp16	64.726	
1IGD	SARS-2-nsp12	59.016	See fig S6A
1IGD	SARS-2-nsp13	57.377	See fig S6B
2FD7	2OHG	54.348	See fig S6C
1IGD	1KMO	52.459	See fig S6D
2FD7	SARS-2-nsp12	52.174	-
SARS-2-nsp12	SARS-2-nsp7	50.633	See fig S6E
2FD7	SARS-2-nsp13	50.000	-
1IGD	2GG4	49.180	-
1JEJ	2FD7	47.826	-
1V2N	2FD7	47.826	-
5NZM	SARS-2-nsp7	46.835	See fig S6F

Supplementary Table 6: Featurization performance. Performance of the featurization scheme used to generate training labels from the simulations above on the same 12 CryptoSite proteins on which it was optimized. Residues within 5Å of the cryptic site ligand in the *holo* crystal structure were used as positive true labels.

	**PR AUC**	**ROC AUC**	**class split**
**4AKE**	0.457	0.848	0.136
**1BSQ**	0.644	0.908	0.099
**1EX6**	0.409	0.838	0.091
**1ALB**	0.552	0.898	0.160
**1NEP**	0.771	0.903	0.185

**1NI6**	0.553	0.900	0.099
**2BLS**	0.384	0.908	0.025
**2QFO**	0.254	0.738	0.097
**3F74**	0.272	0.762	0.111
**1EXM**	0.117	0.708	0.074
**1ADE**	0.417	0.900	0.056
**1MY0**	0.504	0.923	0.070
**mean ± 1 SD**	0.445 ± 0.171	0.853 ± 0.072	0.100 ± 0.044

Supplementary Table 7: FAST simulations. We ran the FAST adaptive sampling algorithm (see methods) on 15 proteins from CryptoSite, 9 highly rigid proteins, and 10 proteins with many *holo* crystal structures in order to generate training data and negative examples for our validation and test sets. The number of FAST rounds and RMSD cluster radius used for simulations of each protein are given in the table below. FAST was run with 10 40 ns long parallel simulations per round for all proteins.

**Protein set**	**PDB ID**	**cluster radius**	**number of rounds of FAST**
Training	1ADE	0.14	5
	1ALB	0.125	5
	1BSQ	0.1	5
	1EX6	0.18	5
	1EXM	0.14	7
	1MY0	0.14	5
	1NEP	0.08	5
	1NI6	0.14	5
	1OFV	0.11	5
	1QYS	0.12	5
	1RHB	0.12	5
	1RTC	0.14	5
	2BLS	0.12	5
	2CM2	0.12	5
	2QFO	0.13	5
	3F74	0.12	5
	4AKE	0.2	5

	5BVL	0.11	5
Highly rigid proteins providing negative examples for the test set	1AMM	0.1	3
	1IGD	0.14	3
	2ALP	0.1	3
	2FD7	0.1	3
	4HJK	0.1	3
	4TQL	0.14	3
Proteins with many *holo* crystal structures providing negative examples for the test set	1BTP	0.1	3
	1HCL	0.14	3
	2OSS	0.15	3
	2ZHV	0.15	3
	3GZ0	0.12	3
	4APE	0.15	3
	5E4P	0.11	3
	5NZ5	0.13	3
	5UOJ	0.17	3
	5X8U	0.12	3
Structures from AlphaFold	Cyclin 1A	0.1	3
	PIM2	0.13	3
	WNT2	0.26	3

Supplementary Table 8: PocketMiner and CryptoSite performance on individual proteins. PocketMiner and CryptoSite were both run on the test set assembled in this work, and the accuracy of the predictions on each protein was calculated. The first set of proteins were known to form cryptic pockets and hence served as source of positive residues. The second set of proteins contained residues which were very unlikely to form cryptic pockets based on experimental and simulation data. Hence, this set of proteins, comprised of hyper-rigid proteins and proteins with extensive ligand co-crystal structures, was used a source of negative residues.

type	***apo* PDB ID**	**CryptoSite accuracy**	**PocketMiner accuracy**
Cryptic pocket	6hb0	0.667	1.000
	2lao	0.667	0.933
	1urp	0.769	1.000
	6ypk	0.875	0.417

	3ugk	0.762	0.976
	5g1m	0.684	0.789
	5nzm	0.889	0.963
	4v38	0.913	1.000
	2w9t	0.850	0.550
	2hq8	0.760	0.600
	4r72	0.625	0.750
	5za4	0.875	0.875
	2cey	0.611	1.000
	1kmo	0.773	0.909
	5nia	0.963	0.926
	2fjy	0.789	0.421
	3p53	0.688	1.000
	1kx9	0.781	0.000
	1tvq	0.857	1.000
	2zku	0.774	0.903
	3nx1	0.636	0.636
	4i92	0.842	0.947
	3qxw	0.650	0.150
	5h9a	0.955	1.000
Highly rigid protein	2fd7	1.000	0.978
	4tql	0.572	1.000
	2alp	0.697	0.787
	1amm	0.770	0.865
Proteins with many holo crystal structures	4ape	0.814	0.873
	5uoj	0.755	0.717
	3gz0	0.686	0.863
	1btp	0.802	0.676
	2oss	0.563	0.850
	5zn5	0.754	0.836
	5x8u	0.732	0.813
	**mean**	**0.766**	**0.800**

Supplementary Table 9: PocketMiner sensitivity by protein topology and cryptic pocket type for test set proteins. Proteins used as sources of negative examples were not included. One protein with no assigned CATH code (PDB ID 6YPK) was excluded from the **CATH class** category and one protein with a large rearrangement (PDB ID 2FJY) was excluded from both the **Direction** and **Motion type** categories.

	**Class**	**Mean**	**Standard deviation**
CATH class	1	0.492	0.394
	2	0.803	0.315
	3	0.895	0.138
Direction	forward	0.677	0.347
	reverse	0.927	0.089
Motion type	Secondary structure change	0.653	0.358
	Interdomain motion	0.944	0.079
	Secondary structure element motion	0.632	0.394
	Loop motion	0.818	0.237
