# Supplementary material for: Predicting locations of cryptic pockets from single protein structures using the PocketMiner graph neural network
Source: Nat Commun. 2023 Mar 1;14:1177. doi: 10.1038/s41467-023-36699-3 (PMC9977097; doi:10.1038/s41467-023-36699-3)
Supplement: Supplementary file 2 — Description of Additional Supplementary Files [file 41467_2023_36699_MOESM2_ESM.docx]

**File Name: Supplementary Data 1**

**Description**: Supplementary data tables containing information about proteins used for training (sheet entitled “training_5fcv”); the proteins contained in the newly curated cryptic pocket dataset (“validation_and_test_sets”); examples of highly rigid proteins with supporting evidence (“highly_rigid_proteins”); examples of proteins that were used to identify residues that do not form cryptic pockets because they had numerous ligand-bound crystal structures (“MOAD negatives”); and the sequence identity between proteins in this study (“sequence_identity”).
